# Application of DenTeach in Remote Dentistry Teaching and Learning During the COVID-19 Pandemic: A Case Study

**DOI:** 10.3389/frobt.2020.611424

**Published:** 2021-01-22

**Authors:** Lingbo Cheng, Maryam Kalvandi, Sheri McKinstry, Ali Maddahi, Ambika Chaudhary, Yaser Maddahi, Mahdi Tavakoli

**Affiliations:** ^1^ College of Control Science and Engineering, Zhejiang University, Zhejiang, China; ^2^ Department of Electrical and Computer Engineering, University of Alberta, Edmonton, AB, Canada; ^3^ Manitoba Dental Association, Winnipeg, MB, Canada; ^4^ College of Dentistry, University of Saskatchewan, Saskatchewan, SK, Canada; ^5^ Rady Faculty of Health Sciences, University of Manitoba, Winnipeg, MB, Canada; ^6^ Department of Research and Development, Tactile Robotics, Winnipeg, MB, Canada; ^7^ Dental Council of India and Indian Dental Association, Mumbai, India

**Keywords:** COVID-19, DenTeach, dental services, teaching and learning model, quantitative assessment

## Abstract

In December 2019, an outbreak of novel coronavirus pneumonia occurred, and subsequently attracted worldwide attention when it bloomed into the COVID-19 pandemic. To limit the spread and transmission of the novel coronavirus, governments, regulatory bodies, and health authorities across the globe strongly enforced shut down of educational institutions including medical and dental schools. The adverse effects of COVID-19 on dental education have been tremendous, including difficulties in the delivery of practical courses such as restorative dentistry. As a solution to help dental schools adapt to the pandemic, we have developed a compact and portable teaching-learning platform called DenTeach. This platform is intended for remote teaching and learning pertaining to dental schools at these unprecedented times. This device can facilitate fully remote and physical-distancing-aware teaching and learning in dentistry. DenTeach platform consists of an instructor workstation (DT-Performer), a student workstation (DT-Student), advanced wireless networking technology, and cloud-based data storage and retrieval. The platform procedurally synchronizes the instructor and the student with real-time video, audio, feel, and posture (VAFP). To provide quantitative feedback to instructors and students, the DT-Student workstation quantifies key performance indices (KPIs) related to a given task to assess and improve various aspects of the dental skills of the students. DenTeach has been developed for use in teaching, shadowing, and practice modes. In the teaching mode, the device provides each student with tactile feedback by processing the data measured and/or obtained from the instructor's workstation, which helps the student enhance their dental skills while inherently learning from the instructor. In the shadowing mode, the student can download the augmented videos and start watching, feeling, and repeating the tasks before entering the practice mode. In the practice mode, students use the system to perform dental tasks and have their dental performance skills automatically evaluated in terms of KPIs such that both the student and the instructor are able to monitor student’s work. Most importantly, as DenTeach is packaged in a small portable suitcase, it can be used anywhere by connecting to the cloud-based data storage network to retrieve procedures and performance metrics. This paper also discusses the feasibility of the DenTeach device in the form of a case study. It is demonstrated that a combination of the KPIs, video views, and graphical reports in both teaching and shadowing modes effectively help the student understand which aspects of their work needs further improvement. Moreover, the results of the practice mode over 10 trials have shown significant improvement in terms of tool handling, smoothness of motion, and steadiness of the operation.

## Introduction

The coronavirus disease 2019 (COVID-19) has been declared as a global pandemic by the World Health Organization (WHO). Globally, as of September 7, 2020, there have been 27,208,206 confirmed cases of COVID-19, including 889,989 deaths (WHO, 2020a). COVID-19 is estimated to have a mortality rate of approximately 4.05%. To slow the spread of COVID-19 at both national and community levels, various measures have been implemented such as COVID-19 testing, contact-tracing and quarantine, social and physical distancing, and international travel bans.

Social and physical distancing measures aim to slow the spread of COVID-19 by stopping chains of transmission of the SARS-CoV-2 virus (WHO, 2020b). Physical distancing measures include maintaining at least 2 m of physical distance between people and the reduction of non-essential personal interactions and reducing contact with potentially contaminated surfaces. Social distancing measures for the general public include flexible work arrangements such as teleworking, distance learning, cancellation of public events to prevent crowding, closure of non-essential facilities and services, local and national movement restrictions, staying-at-home measures, and coordinated reorganization of health care and social services networks to protect hospitals. During the time of the global pandemic, people are encouraged to sustain virtual social connections within families and communities.

COVID-19 social distancing policies led to a widespread lockdown of schools and universities, including dental education institutions (UNESCO, 2020). To a large degree, this has resulted in the extension of the study terms, and deferral of exams and graduation dates. COVID-19 lockdown has exhibited serious repercussions for dental education. While theoretical courses have still been delivered online during the COVID-19 pandemic, the delivery of hands-on courses such as restorative dentistry has been challenging while instructors and students self-isolate at home without access to dental equipment. The duration of this teaching interruption is still uncertain, and dental colleges must keep in mind the possibility of a second or third wave of COVID-19. Hence, it is necessary for dental colleges to look for a reliable and robust, yet inexpensive, solution to ensure the continuation of practical skills in dental education (Solana, 2020).

In this paper, we develop a novel portable teaching-learning platform for remote teaching and learning in dentistry (Maddahi et al., 2020). This new platform, DenTeach, provides an opportunity for dental schools to continue teaching and learning from a remote location (such as a home). This device can fill the existing gap for fully remote or physical-distancing-aware teaching and learning in dentistry. The DenTeach platform consists of an instructor workstation (DT-Performer), a student workstation (DT-Student), advanced wireless networking technology, and cloud-based data storage and retrieval. This platform has high efficiency and is able to procedurally synchronize the instructor and the student with real-time video, audio, feel, and posture (VAFP). As DenTeach is packaged in a small portable suitcase, it can be used anywhere by connecting to cloud-based data to retrieve procedures and performance metrics.

In this paper, we describe the available training and learning models, present the developed DenTeach platform, and demonstrate the feasibility of the DenTeach platform through a case study.

## The State of Dental Education

In health sciences, the use of classroom and hands-on instructions by experts has been a training mechanism of choice for most educational programs. This training mechanism is also called the traditional novice-expert apprenticeship model (Collins et al., 1991). In this traditional model, dental students acquire technical dental skills through years of hands-on training in dental laboratories and clinics and receive supervision and feedback on performance skills. Specifically, mentors conduct a procedure that offers the students the opportunity of observing, then assisting, and finally performing that procedure under the supervision of their mentor. Students learn the nuances of required skills through working on artificial materials, cadaveric organs, animals, and case observations, and receive qualitative feedback on their performance from their mentor (Collins et al., 1991).

However, the traditional novice-expert model cannot be continued due to the continued lockdown of the dental school in the age of the COVID-19 pandemic, as students always require the presence of their mentor to practice and learn the key operation skills in a classroom setting. Additionally, in the field of dentistry, this traditional model is time-consuming, and the training process is slow and lacks quantitative measures to assess aspects of technical skills. As a result, trial and error often constitute a major part of learning psychomotor skills for a student. To provide students with continued learning and training education in times of unprecedented crisis like COVID-19, decreased training hours, and increased training efficiency, there is an increasing demand to develop a portable intelligent teaching-learning platform capable of providing remote teaching and learning delivery and quantitative evaluation of dental performance.

### Remote Teaching and Learning Delivery

The current teaching-learning method involves an instructor to provide visual instructions at a central point in the classroom, while students watch, listen, ask questions, and then imitate tasks. As all dental pieces of equipment are placed in dental schools, students do not have access to the equipment once they leave the classroom. However, if practice units and tools at both the instructor’s and students’ work areas are portable, the teaching and learning can be performed remotely (i.e., while self-isolating at home during the pandemic).

### Dental Performance and Skills Assessment

In order to objectively assess technical dental skills (Schwibbe et al., 2016), it is implicit that one must first be able to measure and study essential aspects of dental performance. One important aspect of instrument handling is the ability to use the instrument (such as the dental handpiece) to effectively, yet safely, accomplish the dental goal. There are several tactile skills that should be understood and learned by students. Most importantly, a student should know how to hold the dental handpiece (orientation and position of the handpiece), comprehend how fast the drill should rotate, perceive the level of vibration produced by the handpiece during the performance of a dental task (acceleration and jerk) and receive adequate alerts once a task is performed improperly. The tactile skills listed above may vary depending on the type of tooth, the region of the oral cavity, or conducted tasks.

### Dental Surgical Simulators

Understanding the tactile skills could be made possible through the incorporation of sensory and actuation systems onto a conventional tool such as a dental rotary handpiece in restorative dentistry.

A device for teaching and training dental treatment techniques has been developed that exerts a force on a tooth, preferably using tools, in order to examine or treat this tooth (Riener and Burgkart, 2013). The mandible or a tooth is coupled to a force measuring device in a manner that enables the forces applied to the tooth to be represented. By using force sensors, the force applied by the dentist is measured and used as a reference signal to be compared with the force applied by the student. Moreover, audible signal patterns are retrieved and audibly displayed utilizing an acoustic display unit such as loudspeakers, which means that screams of pain are played if the calculation shows that the tip of the drill invades the area of the nerve'. Additionally, the position of the force-application point of the tool is localized by means of a navigation system, such as a camera and other optical systems.

In the work of Ranta et al. (2007), a training system has been presented using haptic-enabled simulations of dental procedures to provide the sensorimotor involvement needed for dental training. To provide tactile feedback combined with a realistic visual experience, the system integrates an off-the-shelf haptic stylus interface for simulating the movement and feel of the tooltip with a 3D stereoscopic display. The haptic stylus enables the dental student to orient and to operate simulated dental tools. Working on a virtual model viewed in a stereo display, dental students can use a simulated pick to probe a tooth or a simulated drill to prepare a tooth for cavity repair. The touch feedback is simulated by representing these dental instruments as force-to-a-point tools, which map to haptic simulation procedures executed on a computer workstation that also provides the visual display.

Hayka and Eytan (1997) invented a visual-audio-feeling simulation system for dentistry that comprises a dental handpiece with a drill for drilling a cavity in a tooth. A 3D sensor, attached to the dental handpiece, provides the system with the position and orientation of the drill whereas a data processing unit and a display unit simulate the drill end. The system further controls the flow of compressed air operating the drill, and thus controls the drill’s speed. This imitates the sound and hand-feeling associated when drilling through tooth layers of different hardness.

Kuchenbecker et al. (2017) developed a simulator to educate dental students in caries detection; the simulator allows dental faculty to share, record, and replay the tactile vibrations felt through a dental hand instrument. This simulation approach is assessed by asking experienced dental educators to evaluate the system’s real-time and video playback modes. The simulator uses an accelerometer to sense instrument vibrations and a voice coil actuator to reproduce these vibrations on another tool.

Additionally, the Iowa dental surgical simulator unit focuses on tactile skill development (Johnson et al., 2000). The system consists of three hardware components: a computer, a monitor, and a force feedback device with software. Participants interact with the computer by grasping a joystick or explorer handle attached to the force feedback device. Teeth are displayed on the monitor, and the student can manipulate the joystick or explorer in such a way as to “feel” enamel, healthy dentin, and carious dentin. Different haptic responses are received when the joystick or explorer is manipulated over the appropriate areas of the tooth.

### Virtual Reality and Augmented Reality

In addition to physical devices for dentistry training, some studies (Gal et al., 2011; Bakr et al., 2013; Bakr et al., 2014) also exist on the performance of available dental simulators that use the mechanical properties of teeth to simulate the oral cavity on which dental tasks are conducted. Among the developed dental simulators, the concept of virtual reality (VR) is widely used. As early as the 1990s, the concept of a VR dental training system was introduced to practice cavity preparation (Ranta and Aviles, 1999).

Research has assessed the perception of academic staff members on the realism of the Simodont^®^ haptic 3D-VR dental trainer (Bakr et al., 2013) (MOOG Industrial Group, Amsterdam). This simulator comprises a simulator unit, a panel, a stereo projection, a SpaceMouse, a handpiece, and a projector. The Simodont^®^ courseware developed by the Academic Center for Dentistry in Amsterdam allows a variety of operative dental procedures to be practiced in a virtual oral and dental environment with force feedback. PerioSim© was developed for periodontal simulation, which can simulate three typical operations including pocket probing, calculus detection, and calculus removal (Kolesnikov et al., 2008; Luciano et al., 2009). Forsslund Dental system was developed by Forsslund Systems AB in 2008 to provide VR training for practicing dental drilling and wisdom tooth extraction (Forsslund et al., 2009). Rhienmora et al. (2011) designed a haptic VR crown preparation simulator, which includes a VR environment with haptic feedback for students to practice dental surgical skills, in the context of a crown preparation procedure. An individual dental education assistant (IDEA) used a PHANToM Omni haptic device that allowed for six degrees of freedom (DOF) for position sensing and generated three DOF for force feedback. The virtual dental handpiece was locked to the position and orientation of the haptic stylus (Gal et al., 2011).

A VR dental training system was presented to address limitations and to introduce new techniques such as 1) flexible learning with self-teaching not limited to formal training hours, thus increasing students’ training time and reducing the overall future costs; 2) providing students with the opportunity to gain instant feedback and to practice assessment tasks using similar criteria used by examiners; 3) presenting tooth data as a 3D multi-resolution surface model, reconstructed from a patient’s volumetric data to improve real-time performance; 4) collision detection and collision response algorithms used to handle a non-spherical tool such as a cylindrical one; 5) simulation of tooth surface exploration and cutting with a cylindrical burr by utilizing a surface displacement technique (Rhienmora et al., 2008).

Augmented reality (AR) haptic systems have also been used for dental surgical skills training. In the work of Rhienmora et al. (2010), a dental training simulator utilizing a haptic device was developed based on AR and VR technologies. This simulation utilizes volumetric force feedback computation and real-time modification of the volumetric data to overlay 3D models of the tooth operated on and tools used with the real-world view. The image overlay is delivered through a transparent head-mounted display, which is paired to a haptic device for simulation of virtual dental tools. The system allows dentists to practice using a probe to examine the surface of a tooth, to feel its hardness, and to drill or cut the tooth.

### Quantitative Evaluation

Although a variety of dental surgical simulators for teaching and learning has been developed, the lack of quantitative key performance indices (KPIs) to assess aspects of dental skills is still a significant issue to be addressed. With decreasing operating hours and training resources, there is an increasing demand to improve training efficiency and to provide a quantitative evaluation of dental performance using KPIs.

In order to objectively assess technical dental skills, it is implicit that one must be able to measure and study essential aspects of dental performance (described in *Dental Surgical Simulators*) and quantify KPIs. Currently, in dental schools, dental laboratories, and clinics, this knowledge is often conveyed from the instructor to the apprentices through qualitative instructions, such as “be gentle,” “go deeper” or “push harder”. Quantitative vibrotactile data measured during the performance of dental tasks on human teeth remain largely unavailable. Therefore, in addition to developing advanced intelligent dental simulators to reform the traditional novice-expert apprenticeship model and improve teaching and learning performance, there is a strong demand for systematic quantitative evaluation of dental performance using KPIs. To this end, Wang et al. (2011) developed a haptic-based dental simulator, and preliminary user evaluations on its first-generation prototype have been carried out. Based on the detailed requirement analysis of periodontics procedures, a combined evaluation method including qualitative and quantitative analysis was designed.

Table 1 summarizes several existing commercial dental surgical simulators for teaching and learning and their characteristics. In comparison, the developed DenTeach system in this paper is shown in Table 1 as well.

**TABLE 1:** Comparison of dental simulators.

Simulators	Simodont®	PerioSim®	Forsslund	IDEA	DenTeach
Hardware	-Two projectors -Panel PC -3D glasses -Handpiece and mirror connected to force feedback sensors	-Two computer monitors with a haptic device -Crystal eyes stereo glasses™ and a crystal eyes workstation™ -A PHANToM haptic device with 3 DOFs - VR William’s periodontal probe or periodontal explorer	- Polhem haptic device - Kobra oral surgery simulator with two screens - 3D glasses	A stylus with six DOFs position sensor and three DOFs force sensor attached to a stand PHANToM omni	- Two computer monitors with a haptic device - Handpiece connected to a custom-designed sensor
Software	Moog Simodont^®^ dental trainer courseware software	Modified version of Ghost™	Kobra simulation software	ManualDexterity™, caries detection, scaling and Root-Planning™, OralMed™ and PreDenTouch™	DT-performer software
Ability to use off campus	No	Yes	No	Yes	Yes
Feedback sensory channels	Haptic-visual-auditory	Haptic-visual-auditory	Haptic-visual	Haptic-visual	Haptic-visual-auditory
Immediate feedback	No	No	Yes	Yes	Yes
Display type	3D	3D/AR	3D/VR	Monitor screen	Monitor screen
Haptic device	Moog haptic master	PHANToM desktop	PHANToM omni/desktop	PHANToM omni	Custom-designed DT-RealFeel drill
Virtual drilling control	Foot pedal	No	Foot pedal	NA	Foot pedal
Sensor	Force sensor	Force sensors	NR	Position and force sensors	DT-RealFeel sensor
Automatic evaluation	Yes	Yes	Yes	Yes	Yes
Direct transfer data to tutor	Yes	No	No	Yes	Yes
Expert’s database	No	Yes	Yes	No	Yes
Haptic-visual collocation	Yes	No	Yes	Yes	Yes
Practice/test simulation	Yes	Yes	Yes	No	Yes

### DenTeach System

**Figure 1:**
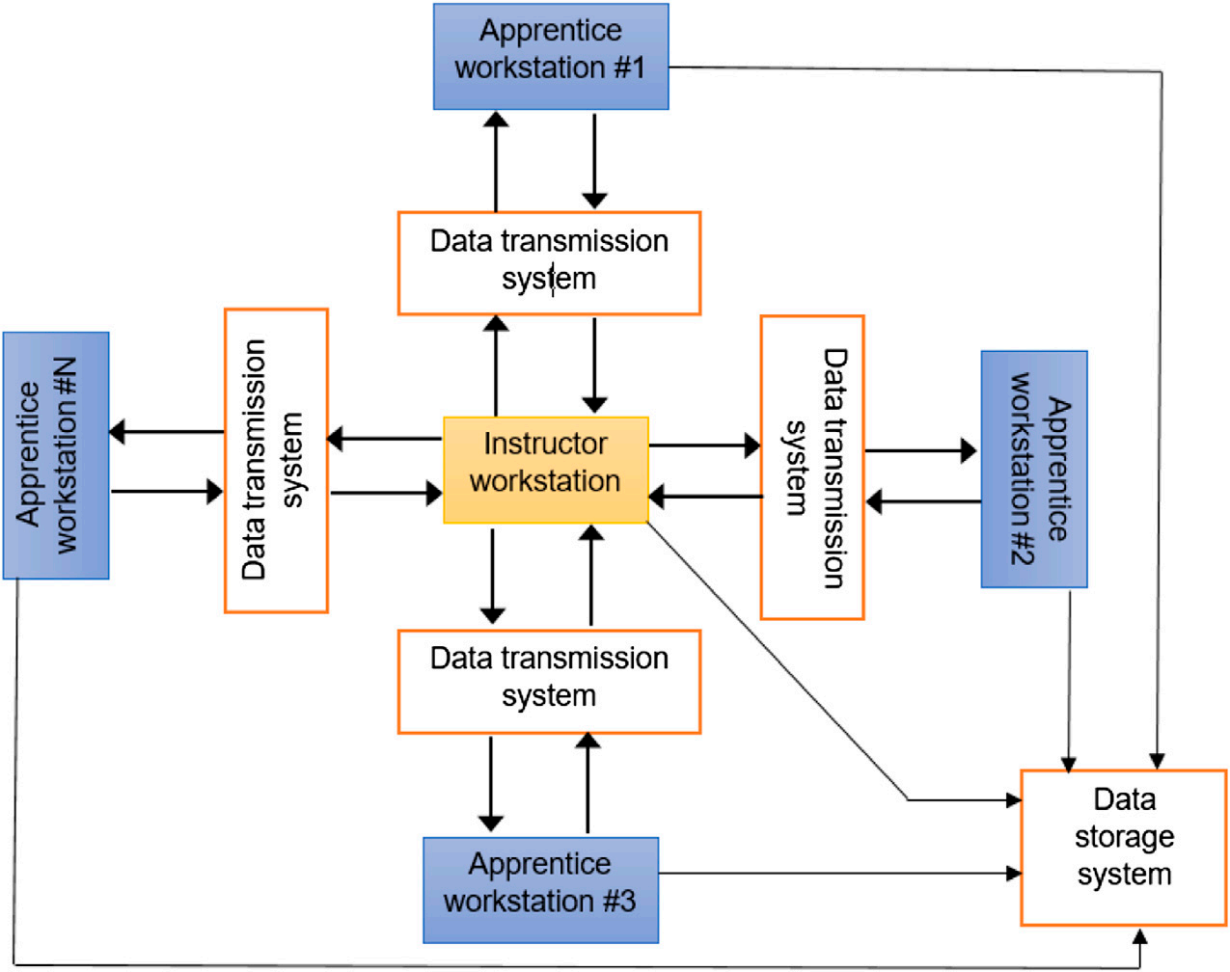
The overall scheme of the DenTeach system comprising an instructor workstation, a number of student workstations, a data transmission system, and a data storage system along with the overall workflow of the main components of the device.

The newly developed portable teaching-learning platform, DenTeach, complements traditional methods and is based on the latest industry technologies including smart sensors, advanced robotics, big data analysis, 3D printing, AR, and cloud-based computing. The system creates a real-life traditional teaching-learning experience by synchronizing an instructor and a student with real-time VAFP. The DenTeach portable platform consisting of a DT-Performer (Instructor’s software), a DT-Student software (Student’s software), advanced wireless networking technology, and cloud-based data storage and retrieval has been developed for use in teaching, shadowing, and practice modes. The data storage system stores VAFP data of the DT-Performer and the DT-Students in both modes, as well as KPIs, defined for evaluating students’ performance. Figure 1 provides an overall scheme of the system. An instructor workstation comprises a commercially available dental handpiece equipped with a wireless sensory system and a video recording system while each student workstation consists of a custom-made haptic-enabled dental handpiece augmented by another sensory system and an actuation system and a video recording system. There are processing systems and display units at each workstation, and a data transmission module to transfer commands between workstations through the cloud.

### Physical Setup

**Figure 2:**
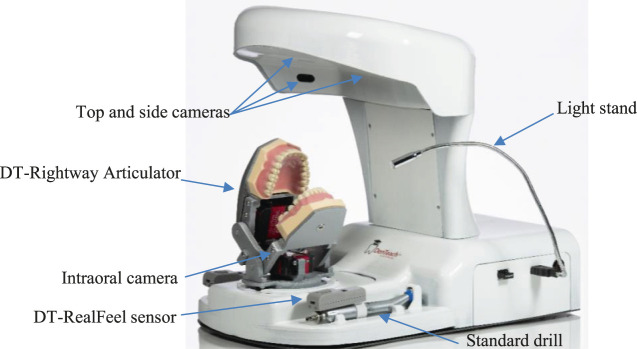
Instructor workstation includes a dental unit, a rheostat, a processing unit, a tooth physical model, a dental handpiece, a set of sensory systems to measure vibrotactile data of the dental handpiece, a sensor to measure rheostat data, an audiovisual recording system, a software, and a display.

**Figure 3:**
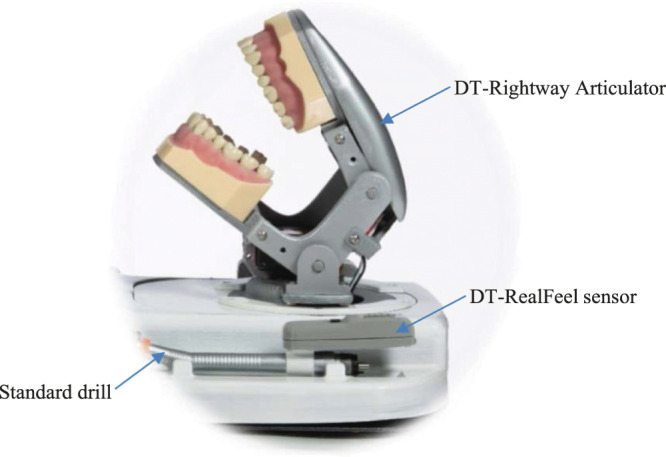
DT-Rightway Articulator and DT-RealFeel sensor attached to a standard dental drill.

DenTeach complements the traditional instructor and student working area by integrating into the existing working setup (which consists of a tabletop, dental unit, and dental instruments). For the instructor work area (Figure 2), the DenTeach platform integrates into a standard instructor work area and dental unit, and consists of DT-Performer software, DT-Rightway Articulator, DT-RealFeel sensors, and four mini cameras. Specifically, the DT-Performer software provides a full classroom view and selectable student profile and performance index. The DT-Rightway Articulator shown in Figure 3 is a custom-designed system that supports upper and lower typodonts. The sensors are wirelessly attached to the standard dental drills to measure quantitative performance data. Each sensor is a state-of-the-art wireless sensor that records and streams the instructor’s hand motion data to the cloud (recorded data will then be imported to each student workstation). DT-Performer interprets data in a real-time fashion and provides advanced statistical data analysis to quantitatively score students’ performance. During each test, the orientation data and dynamic information are measured or calculated that include roll (axial), pitch (back-to-front) and yaw (side-to-side) angles, linear accelerations (3 DOFs), angular accelerations (3 DOFs), angular velocity (3 DOFs), jerk components (3 DOFs), and several KPIs.

To display and record the instructor’s hand operation during teaching procedures, four mini cameras show the top view, two side views, and inside view. All videos are transmitted simultaneously onto the students’ workstations. Additionally, DT-Performer software allows the instructor to select, record, and play over 30 psychomotor performance metrics to objectively measure effort, speed, accuracy, and learning curve.

**Figure 4:**
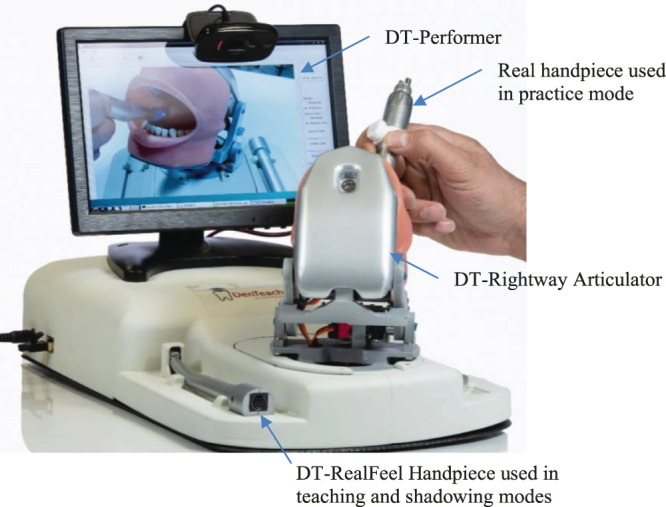
The components of the DT-Student (apprentice workstation): DT-Rightway Articulator, DT-RealFeel Drill, and a monitor.

For the student work area (Figure 4), the DT-Student consists of a fully integrated system with four selectable instructor videos, a student’s drill model superimposed over the videos of the instructor’s drill to enable effective imitation or mimicking, two typodonts affixed to the DT-Rightway Articulator, a student DT-RealFeel Handpiece synchronized to the instructor’s movements while in teaching mode, and a DT-Student software that allows the student to select, record, and play recordings that demonstrate over psychomotor performance metrics to objectively measure effort, speed, accuracy and learning curve. To be more specific, the custom-designed DT-RealFeel Handpiece has a handle grip associated with its components including an actuation system to generate a vibrotactile feeling, a vibrator to apply an abrupt force to the student’s hand as an alarm, and a set of sensory systems along with the data communication system. Besides, the processing unit of the Student’s workstation is arranged to calculate a plurality of different performance indices in which each index is calculated using one or more operating characteristics detected by the sensory system of the DT-RealFeel Handpiece. Similar performance characteristics are calculated using the data from the DT-Performer at the instructor’s station.

### Education Modes

DenTeach allows learning activities in three modes namely teaching, shadowing, and practice.

#### Teaching Mode

In the teaching mode, similar to a general traditional teaching and learning mode, an instructor conducts dental tasks in the instructor’s workstation and students mimic the tasks in the students’ workstations. The main difference is that the DenTeach device uses a data transmission system to provide each student with tactile feedback by processing the data measured and/or obtained from the dental tool of the instructor’s workstation. This helps students understand and perceive how their instructor is conducting the dental operation and tasks without their presence at the instructor’s workplace. Moreover, the data storage system saves information such as data of sensory systems from the instructor and students’ workstations as well as audiovisual recordings taken from the instructor’s workstation. This information can later be retrieved and used for various purposes, for example by students in the practice mode or by instructors for evaluation of student’s performance in both teaching mode and practice mode.

**Figure 5:**
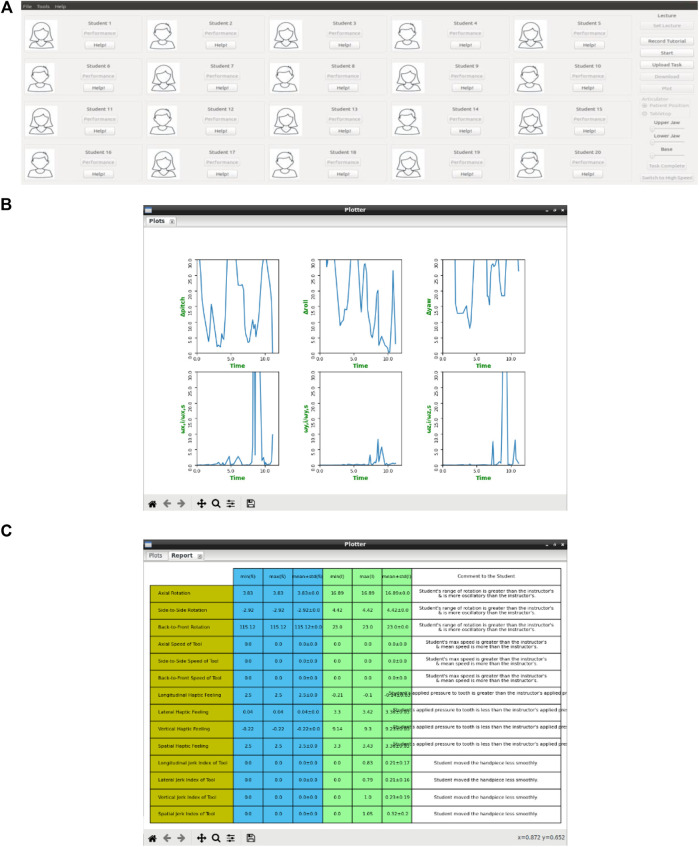
The DT-Performer components and workflow in the teaching mode. **(A)** screenshot of the graphical user interface on the instructor's display unit. Each student can upload their photo to the system. Plotter is a feature that presents the KPIs in form of graphs **(B)** or tables **(C)**.

A dental task is conducted by an instructor using a dental tool on a DT-Rightway Articulator. The instructor processing unit running DT-Performer software includes the main processor responsible for: 1) receiving and analyzing sensory systems data recorded during performance of a dental task by the instructor; 2) recording video and audio that are taken from the audiovisual recording system; 3) communicating with the students’ workstations and the data storage system via the data transmission system; and 4) providing the instructor with user-friendly software designed for teaching different dental tasks that are screened on the display (see Figure 5).

The DT-Performer software enables the instructor to choose different options including the teaching session along with the time and date as well as the type of the dental task. Each set of students’ KPIs is displayed graphically on the screen located at the instructor's workstation, which helps the instructor monitor student performance during a teaching session (Figures 5A and 5B). Additionally, the software can authenticate each student’s access request when they enter the physical/online classroom.

**Figure 6:**
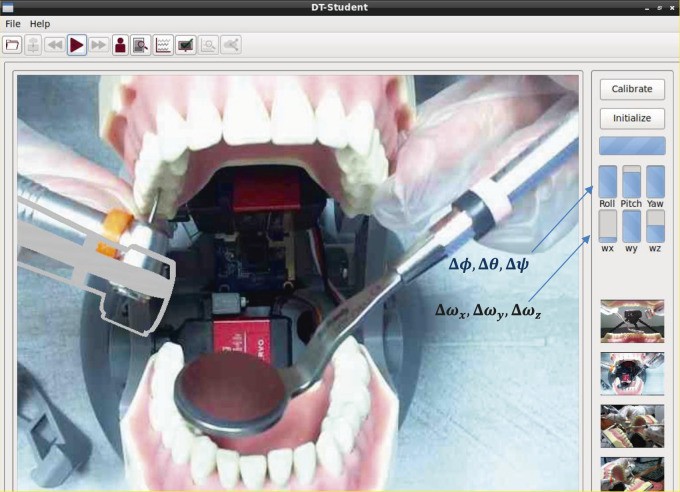
The DT-Student components and workflow for teaching purposes. In addition to plotting the performance indices, the DT-Student allows students to switch between the four views streaming from the instructor workstation and choose the most discernable view to learn. A 3D model of the student's handpiece (DT-RealFeel Handpiece) is superimposed to the videos streaming from the instructor workstation and allows each student to visually monitor their tool handling. If the student’s movement is not within the acceptable range of motion, in addition to the bar charts, the alarm inside the DT-RealFeel Handpiece will start vibrating to help the student to stay on track.

In Figure 6, different components used in students’ workstations are illustrated. A student holds a custom-designed DT-RealFeel Drill on a DT-Rightway Articulator, the same as the model used in the instructor’s workstation. The DT-RealFeel Drill and DT-Rightway Articulator are mounted onto a platform for initialization and registration purposes. The student processing unit runs the DT-Performer software and provides each student with a user-friendly interface designed for the teaching mode.

In teaching mode, the student processing unit is responsible for: 1) receiving and analyzing data of the sensory system located inside the custom-designed training tool; 2) communicating with both instructor’s workstation and the data storage system via the data transmission system; 3) generating control inputs for the vibrotactile actuation system and the vibrator that are located inside the custom-designed training tool, based on data received from the instructor’s workstation through the data transmission system; 4) displaying video and audio recordings, which includes the instructor’s hand, tool and tooth physical model received from the instructor’s workstation through the data transmission system in real-time; 5) superimposing 3D model of the custom-designed training tool onto the video in an AR environment screened on the display (see the inset in Figure 6), and moving the 3D model using processed data of sensory system; 6) calculating KPIs for evaluation of each apprentice’s performance during the teaching session based on the data taken from the sensory systems; 7) sending KPIs of each student to the instructor’s workstation and data storage system via the data transmission system.

The DT-Student software of the student workstation enables each student to get access to data taken from the DT-Performer during the dental operation. Moreover, the student software helps the students monitor their own KPIs during the teaching session and receive detailed statistical reports on how well they could follow the dental task in teaching mode.

There are two factors that students can continuously monitor during the teaching sessions; these factors are plotted in a real-time fashion using 6-bar charts in DT-Performer, as seen in Figure 6.

(1) Tool handling ability is determined by the acceptable deviation set by the instructor. At the beginning of the experiment, the instructor sets the acceptable amount of the student’s deviation to be less than 15° for the roll (ϕ), pitch (θ), and yaw (ψ) angles. Deviations are calculated by subtracting the angle of the student's tool from the corresponding angle of the instructor tool as follows:

$$\text{Δ}\phi =\left|\phi_{instructor}-\phi_{student}\right|$$

$$\text{Δ}\theta =\left|\theta_{instructor}-\theta_{student}\right|$$ (1)

$$\text{Δ}\psi =\left|\psi_{instructor}-\psi_{student}\right|$$

(2) The smoothness of the motion or student’s ability to move the tool at the same speed as the instructor, which is defined as:

$$\text{Δ}\omega_{x}=\frac{\omega_{x,instructor}}{\omega_{x,student}}$$

$$\text{Δ}\omega_{y}=\frac{\omega_{y,instructor}}{\omega_{y,student}}$$ (2)

$$\text{Δ}\omega_{z}=\frac{\omega_{z,instructor}}{\omega_{z,student}}$$

where $\omega_{x}$, $\omega_{y}$, and $\omega_{z}$ indicate the angular velocities about x, y, and z, respectively. Note that when the student is not performing the dental task as smoothly as the instructor (or moves the tool faster than the instructor) the numerator becomes much smaller than the denominator and the ratio will be close to zero, while the ratio of one means that the student is able to handle the tool as smoothly as the instructor.

#### Shadowing Mode

In shadowing mode, a student can download augmented videos (4 videos from the class session along with signals of sensory systems and values of KPIs) and start watching, feeling, and repeating the task before entering the practice mode. In the shadowing mode, a student uses the DT-RealFeel handpiece to shadow dental tasks taught by the instructor. In this operating mode, the video of the dental task - that has already been performed by the instructor–is displayed on the DT-Student monitor while superimposing a 3D model of the training tool (DT-RealFeel handpiece) onto the video, in an AR environment, when rehearsing the dental task.

#### Practice Mode

In practice mode, the setup components for a student are the same as the ones described in Figure 6 except the training tool, which is the same as the dental tool used by the instructor in the teaching mode instead of the DT-RealFeel Handpiece. In practice mode, a student processing unit is responsible for: 1) receiving and analyzing data of sensory systems; 2) communicating with the data storage system via data transmission system and receiving sensory data already stored by the instructor during the teaching session and 3) calculating student’s KPIs based on both data taken from sensory systems and data from instructor’s workstation.

While a student is performing a dental task in practice mode, the DenTeach software displays KPIs of a student graphically. The software also generates statistical and graphical performance reports for dental tasks performed by a student in practice mode. These performance reports are uploaded to the data storage system via the data transmission system, and are made available to the instructor for evaluation purposes.

### KPIs

In addition to the qualitative assessment of dental skills conducted by an instructor, the performance of each student is assessed quantitatively individually and comparatively. For quantitative evaluation, two sets of KPIs are used.

Table 2 shows the signals that are recorded and shown during the performance of the dental task that enable the student and the instructor to assess the performance of the student throughout teaching (24 KPI signals), shadowing (24 KPI signals), and practice modes (48 KPI signals). Each KPI signal is meant to assess a specific skill of the student that includes: 1) assessment of the effort put in by the student; 2) assessment of the smoothness factor of the student’s tool handling skill; 3) assessment of haptic feeling, *i.e.*, pressure applied to the tooth; and 4) assessment of the steadiness factor of the student’s tool handling skill. Table 3 lists the second set of KPIs summarizes statistical indices of the signals presented in Table 2 in an enumerative manner. Using the information provided by this set of KPIs, each student (and the instructor) can have an inclusive summary of the student’s dental skills during teaching (40 PKIs), shadowing (40 PKIs), or practice (82 PKIs) modes. These numbers are also calculated for a task conducted during every trial; therefore, the student is able to monitor their progress over multiple trials.

**TABLE 2:** Performance measures and KPI signals.

Mode		KPI signal	Student	Instructor	Difference	Assessment purpose
Practice (48)	Teach and shadow (24)	Tool handling angulation • Axial rotation of the tool • Side-to-side rotation of the tool • Back-to-front rotation of the tool • Overall tool handling skill	√ √ √ √	√ √ √ √	√ √ √ √	Assessment of the effort put by the student
						12 KPI signals in total
		Tool handling smoothness • Axial speed of the tool • Side-to-side speed of the tool • Back-to-front speed of the tool • Overall smoothness in tool handling	√ √ √ √	√ √ √ √	√ √ √ √	Assessment of the smoothness of student’s tool handling skill
						12 KPI signals in total
		Haptic sensation • Longitudinal haptic feeling • Lateral haptic feeling • Vertical haptic feeling • Spatial haptic feeling	√ √ √ √	√ √ √ √	√ √ √ √	Assessment of haptic feeling, i.e., the pressure applied to the tooth
						12 KPI signals in total
		Tool handling steadiness • Longitudinal jerk index of the tool • Lateral jerk index of the tool • Vertical jerk index of the tool • Spatial smoothness in tool handling	√ √ √ √	√ √ √ √	√ √ √ √	Assessment of the steadiness of student’s tool handling skill
						12 KPI signals in total

**TABLE 3:** Performance measures and KPI numbers.

Mode		Characteristics	Minimum	Maximum	Range	Average	Standard deviation	Purpose
Practice (82)	Teach and shadow (40)	Tool handling angulation • Axial rotation of the tool • Side-to-side rotation of the tool • Back-to-front rotation of the tool • Overall tool handling skill	√ √ √ √	√ √ √ √	√ √ √ √	√ √ √ √	√ √ √ √	Assessment of the effort put by the student
								20 KPIs in total
		Tool handling smoothness • Axial speed of the tool • Side-to-side speed of the tool • Back-to-front speed of the tool • Overall smoothness in tool handling	√ √ √ √	√ √ √ √	√ √ √ √	√ √ √ √	√ √ √ √	Assessment of the smoothness of student’s tool handling skill
								20 KPIs in total
		Haptic sensation • Longitudinal haptic feeling • Lateral haptic feeling • Vertical haptic feeling • Spatial haptic feeling	√ √ √ √	√ √ √ √	√ √ √ √	√ √ √ √	√ √ √ √	Assessment of haptic feeling, i.e., the pressure applied to the tooth
								20 KPIs in total
		Tool handling steadiness • Longitudinal jerk index of the tool • Lateral jerk index of the tool • Vertical jerk index of the tool • Spatial smoothness in tool handling	√ √ √ √	√ √ √ √	√ √ √ √	√ √ √ √	√ √ √ √	Assessment of the steadiness of student’s tool handling skill
								20 KPIs in total
		Task completion time	√ (1 index)					Performance time
													1 KPI in total
		Interruption index	√ (1 index)					Continuous motion
													1 KPI in total

## Case Study

### Experimental Setup

**Figure 7:**
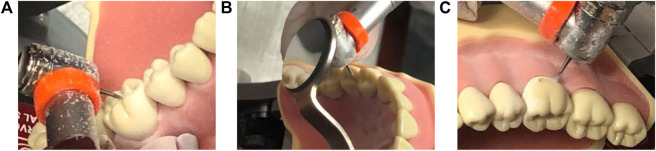
Screenshots of the dental tasks - **(A)** Task 1: Class I composite preparation on tooth #46; **(B)** Task 2: Class II composite preparation on tooth number 45; **(C)** Task 3: Class V composite preparation on tooth number 46.

DenTeach was used to measure the KPIs and the ability of the system to help an instructor and students teach and learn more effectively compared to existing traditional techniques. Plastic teeth were mounted onto the typodonts inside the DT-Rightway Dental Articulator. Three common dental tasks were completed by an experienced dentist as the instructor (MK), while a student (AM) mimicked the performance of dental tasks at the student workstation. The instructor used the sensor modified dental handpiece to perform a Class I, II, or V composite preparation, which involves different lesion sizes and caries, over an interval of active practicing on a plastic tooth that is characterized by rheostat engagement and drill operation. More detailed information on the procedures is given in Tam’s work (Tam, 2020). A screenshot of the tasks is shown in Figure 7.

### Teaching Mode

**Figure 8:**
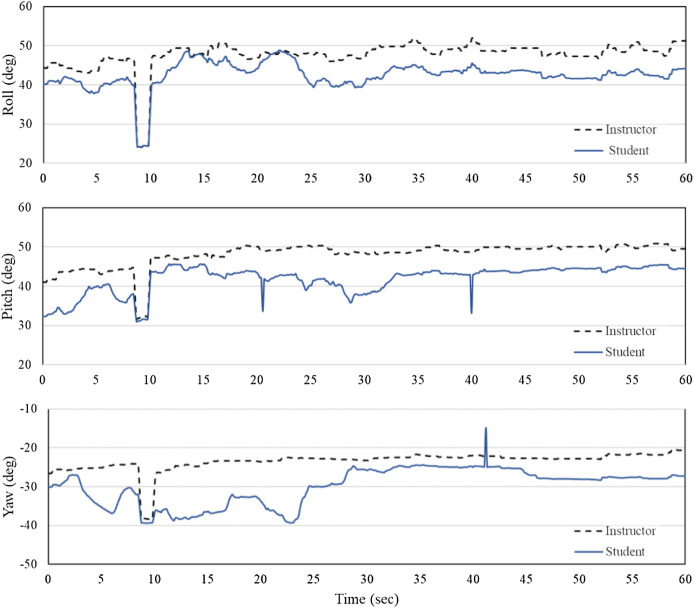
Angulations of the instructor (blue line - solid) and the student’s (black line - dashed) handpiece while performing task 1 over a typical time interval of 60 s - roll (ϕ), pitch (θ), and yaw (ψ).

**Figure 9:**
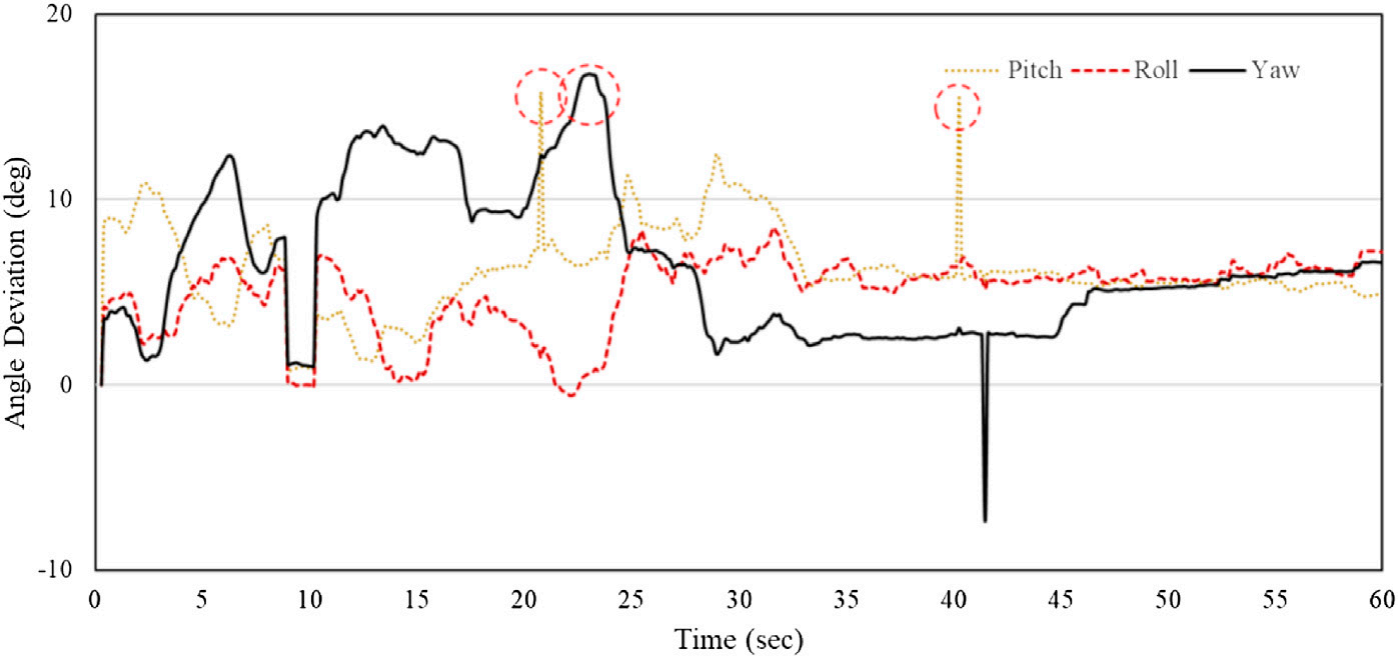
Deviation of the student’s tool handling from the instructor (roll: Δϕ, pitch: Δθ and yaw: Δψ) while performing Task 1 over a typical time interval of 60 s.

During the experiments, the outputs of the DT-Performer and DT-Student software were exported in this section. The software recorded, analyzed, and plotted real-time data from the instructor’s dental handpiece. Figure 8 depicts three Euler angles of the handpiece held by the instructor and the student as well as the deviations between their angulations, for Task 1. As observed in Figure 8, the instructor’s motion was followed reasonably well by the student that held the DT-RealFeel Handpiece, as the student’s motion deviations are within *a range expected by the instructor* (15 degrees of deviation). The amount of the deviation could change once the students become more experienced or if the instructor changes the deviation range. For this typical interval, the amount of angle deviation for roll angle was within the acceptable interval set by the instructor for most parts of the performance of the task, as depicted in Figure 9. However, a deviation of more than 15° was recorded 3 times during the teaching mode, one for the yaw angle and two for the pitch angle that accordingly received an excessive vibration signal reminding the student to keep the handpiece within the allowable zone. The number of deviations for Task 2 and 3 were 2 and 4, respectively. The student took the handpiece back to the allowable range once the excessive signal was generated by the RealFeel handpiece. We expect to observe a decreased amount of deviation once the student is familiar with dental tasks, as shown in experiments.

Tables 4–6 show the values of select KPIs that are reported for the instructor and the student after the completion of Tasks 1, 2, 3, respectively. A combination of the KPIs, video views and graphical reports in both teaching and shadowing modes help the student understands which aspects of the work need further improvement. For example, in all KPIs reported, the standard deviation of the student is larger than the instructor’s indicating that the student is required to work on the skill of tool handling (axial rotation, back-front motion, and side to side motion) and the speed of tool handling (axial rotation steadiness, back-front motion steadiness, side to side motion steadiness, and overall motion steadiness). The shaded cells in Table 5 show that the student was out of range in terms of the tool handling and an alarming signal was applied to the handpiece to bring the hand back on the track.

**TABLE 4:** Student and instructor KPIs while performing Task 1 in teaching mode.

Characteristics	Student					Instructor				
	Min	Max	Ran	Ave	Std	Min	Max	Ran	Ave	Std
•Axial rotation of the tool (ϕ) - deg	24.12	48.87	24.75	42.59	3.50	24.11	52.02	27.92	47.62	3.81
•Side-to-side rotation of the tool (ψ) - deg	−39.44	−14.75	24.69	−29.91	4.70	−38.31	−20.39	17.92	−23.42	2.48
•Back-to-front rotation of the tool (θ) - deg	30.97	45.72	14.75	41.91	3.53	31.73	51.01	19.28	48.10	3.17
•Axial speed of the tool ($\dot{\phi }$) - deg/s	0.05	13.33	13.28	2.16	1.58	−3.36	2.22	5.58	−0.23	3.47
•Side-to-side speed of the tool ($\dot{\psi }$) - deg/s	0.02	7.75	7.73	1.45	1.03	−1.75	1.64	3.39	−0.31	1.61
•Back-to-front speed of the tool ($\dot{\theta })$ - deg/s	0.01	15.20	15.19	1.64	1.91	−1.34	1.38	2.72	0.15	1.77

**TABLE 5:** Deviation of the student’s KPIs from the instructor’s KPIs while performing Tasks 1, 2, and 3 in teaching mode.

Characteristics	Task 1					Task 2					Task 3				
	Min	Max	Ran	Ave	Std	Min	Max	Ran	Ave	Std	Min	Max	Ran	Ave	Std
Tool handling angulation															
• Axial rotation of the tool (Δϕ) - deg	−0.56	8.54	9.10	5.03	2.02	−0.76	2.13	2.90	2.01	2.12	−0.58	1.37	1.94	1.83	2.06
• Side-to-side rotation of the tool (Δψ) - deg	−7.38	16.82	24.19	6.49	4.02	−1.84	33.63	35.47	5.19	3.24	−0.18	17.17	17.36	5.31	1.77
• Back-to-front rotation of the tool (Δθ) - deg	0.77	15.80	15.04	6.20	2.28	0.19	4.74	4.55	10.22	2.16	0.22	2.13	1.91	17.38	1.84
Tool handling smoothness															
• Axial speed of the tool (Δϕ) - deg/s	0.05	13.33	13.28	2.16	1.58	0.04	8.53	8.49	1.19	1.91	0.05	12.88	12.83	0.83	3.05
• Side-to-side speed of the tool ($\Delta \dot{\psi }$) - deg/s	0.02	7.75	7.73	1.45	1.03	0.03	3.33	3.31	1.71	1.47	0.04	1.43	1.39	0.84	0.72
• Back-to-front speed of the tool ($\Delta \dot{\theta }$) - deg/s	0.01	15.20	15.19	1.64	1.91	0.00	13.83	13.83	1.68	2.95	0.01	12.17	12.17	2.44	3.74

**TABLE 6:** Student’s KPIs quantified while performing Task 1 in shadowing mode over trials 1 and 5.

Characteristics	Trial 1					Trial 5				
	Min	Max	Ran	Ave	Std	Min	Max	Ran	Ave	Std
Tool handling angulation										
• Axial rotation of the tool (ϕ) - deg	25.81	53.51	27.70	44.93	3.63	23.73	36.90	13.18	36.89	3.24
• Side-to-side rotation of the tool (ψ) - deg	−43.19	−14.90	28.29	−31.40	4.82	−34.58	−13.62	20.96	−24.48	4.64
• Back-to-front rotation of the tool (θ) - deg	31.28	45.95	14.67	42.96	3.56	31.17	29.01	2.16	40.95	3.39
Tool handling smoothness										
• Axial speed of the tool ($\dot{\phi }$) - deg/s	0.05	13.53	13.48	2.18	1.72	0.05	12.79	12.74	2.20	1.29
• Side-to-side speed of the tool ($\dot{\psi }$) - deg/s	0.02	8.02	8.00	1.52	1.04	0.02	5.31	5.29	1.22	0.96
• Back-to-front speed of the tool ($\dot{\theta })$ - deg/s	0.01	15.28	15.27	1.69	1.95	0.01	10.03	10.03	1.74	1.50

### Shadowing Mode

In shadowing mode, the student used the RealFeel handpiece to review the tasks taught by the instructor. In addition to acquiring more quantitative feedback on the tasks, this mode helps the student become confident and prepare for the practice mode to get hands-on practice with the actual dental handpiece. One advantage of the shadowing mode is to save material and time, with minimal supervision. Therefore, the student is not restricted to academic labs for extended hours, as the portable and compact unit can be used anywhere to practice dental operations over the Internet.

In this case study, the student performed five trials of task 1 in shadowing mode. This was assessed in terms of the three ϕ, θ, ψ angles and possible deviations from the instructor’s angulation were monitored as well as the amount of the pressure to be exerted on the tooth 46. The results of the KPIs are presented in Table 6 for trial 1 and trial 5. As observed, the range of motion in the last trials (#5) with respect to the first trial (#1) along axial, side-to-side, and back-to-front rotations decreased by 52.4%, 25.9%, and 74.9%, respectively. Moreover, the standard deviations in both angulation and speed components were reduced from trial 1 to trial 5, which shows that the improvement in student’s ability to handle the tool in a more limited workspace and a smoother manner using the DenTeach setup. For example, the standard deviation of axial rotation changed from 3.63 to 3.24.

### Practice Mode

In practice mode, the student used the actual dental handpiece to practice the three tasks. For this mode, a wireless sensory system that is identical to the sensors used by the instructor is used to measure the signals used for calculating the KPIs. The sensory system and camera then recorded and communicated the audiovisual vibrotactile information to the database to be compared with those of the instructor. Therefore, the student is able to submit the results of each trial to the instructor along with the audiovisual signals at the end of each trial. The KPIs of the first (#1) and last (#10) practice trials are listed in Table 7. As observed, the student improved the scores in most of the KPIs including the haptic jerk index that is used for assessing the steadiness of tool handling. Specifically, the maximum value of longitudinal, lateral, and vertical jerk indices decreased by 1.3%, 64.8%, and 25.8%, respectively, indicating the increase in the steadiness of hand’s motion from the first trial to the last trial.

**TABLE 7:** KPIs quantified while performing Task 1 in practice mode over the first and the last trials (1 and 10).

Characteristics	Trial 1					Trial 10				
	Min	Max	Ran	Ave	Std	Min	Max	Ran	Ave	Std
Tool handling angulation										
• Axial rotation of the tool (ϕ) - deg	22.67	79.16	56.49	22.67	79.16	24.72	51.06	26.34	24.72	51.06
• Side-to-side rotation of the tool (ψ) - deg	−63.11	−15.93	47.17	−63.11	−15.93	−41.22	−15.34	25.87	−41.22	−15.34
• Back-to-front rotation of the tool (θ) - deg	30.25	72.24	41.99	30.25	72.24	31.90	47.78	15.88	31.90	47.78
Tool handling smoothness										
• Axial speed of the tool ($\dot{\phi }$) - deg/s	0.06	16.80	16.74	0.06	16.80	0.05	13.66	13.62	0.05	13.66
• Side-to-side speed of the tool ($\dot{\psi }$) - deg/s	0.03	13.18	13.14	0.03	13.18	0.02	7.83	7.81	0.02	7.83
• Back-to-front speed of the tool ($\dot{\theta })$ - deg/s	0.01	23.10	23.10	0.01	23.10	0.01	15.43	15.42	0.01	15.43
Haptic sensation										
• Longitudinal haptic feeling - deg/s^2^	9.36	−5.80	15.16	0.16	7.73	9.11	−6.40	15.51	−0.74	7.18
• Lateral haptic feeling - deg/s^2^	−1.12	−8.88	7.76	0.18	5.20	−2.12	−8.98	6.86	0.03	4.20
• Vertical haptic feeling - deg/s^2^	−1.12	−5.80	4.68	−3.27	0.81	−1.47	−6.05	4.58	−3.77	−0.04
Tool handling steadiness										
• Longitudinal jerk index of the tool - deg/s^3^	−7.14	7.63	14.77	0.02	1.01	−6.39	7.53	13.92	−1.63	0.64
• Lateral jerk index of the tool - deg/s^3^	−2.61	2.16	4.77	0.00	0.59	−1.81	0.76	2.57	−1.20	0.40
• Vertical jerk index of the tool - deg/s^3^	−2.75	2.52	5.27	0.00	0.10	−1.55	0.32	1.87	−0.80	−0.06
Task completion time - s	125					95.56				
Interruption index	16					9				

**Figure 10:**
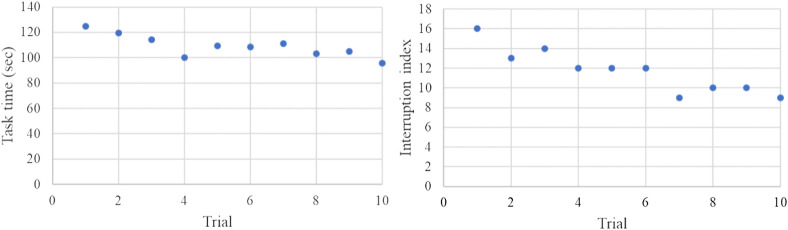
Variations of the task completion time and the interruption index over 10 trials in the practice mode. The mean values and standard deviations of task completion time and interruption indices are 109.13 + 8.88 and 11.7 + 2.26, respectively.

Figure 10 shows the variations of the task completion time and the interruption index (the number of rheostat engagements and disengagements). As observed, after 10 trials, the student could complete the task 25.5% faster than the first trial; however, the interruption index was improved by 43.7% showing that the student was more confident in handling the handpiece in the last trial compared to the first trial. The task completion time showed a mean ± standard deviation (std) of 109.13 + 8.88 and the interruption indices had a mean ± std of 11.7 + 2.26.

## Conclusion

The COVID-19 pandemic response has resulted in remote and physical distancing restrictions to limit the spread and transmission of the novel coronavirus. This has caused significant adverse effects on dental education (i.e. difficulties in the delivery of practical courses such as restorative dentistry and deferral of exams). To help dental institutions continue delivering education remotely, a compact and portable teaching-learning platform, DenTeach, has been developed for remote teaching and learning. The platform includes an instructor workstation (DT-Performer), a student workstation (DT-Student), advanced wireless networking technology, and cloud-based data storage and retrieval. By providing real-time video, audio, feel, and posture (VAFP) information, the platform synchronizes the operations of the instructor and the student. Besides, the platform can provide quantitative KPIs of the student to both the instructor and the student to evaluate the student’s skill level.

DenTeach follows and expands on the traditional novice-expert apprenticeship model of instruction to enhance dental training programs. It has been developed for use in teaching, shadowing, and practice modes. In teaching mode, the student can perceive how the instructor is conducting the dental operation through tactile feedback obtained from the dental tool of the instructor's workstation. In shadowing mode, the student can watch, feel, and repeat the tasks alone by downloading the augmented videos. In practice mode, students can use the system to perform dental tasks and have their dental performance skills automatically evaluated in terms of KPIs. A case study was performed to demonstrate the feasibility of the device, and the results show that a combination of KPIs, video views, and graphical reports in both teaching and shadowing modes can effectively help the student understand which aspects of their work need further improvement.

DenTeach is a useful invention for pedagogical and professional purposes, which can be used for training and educating students in both clinical/laboratory and remote (*i.e.*, home) settings due to its compact and portable size. This device facilitates both fully remote and physical-distancing aware teaching and learning in dentistry. Additionally, the DenTeach platform can be useful during the pandemic recovery phase, when dental schools are allowed to return to normal operations. Once dental schools are reopened, there will be a surge in teaching, practicing, and exams. DenTeach can be used to increase the efficiency of the training process, thus allowing dental schools to clear the backlog of activities faster. Before the second wave of COVID-19 hits, decision-makers at dental colleges may want to ensure they have adequate resources to continue teaching and testing from a remote location and minimize the backlog of deferred activities. DenTeach can be used as an effective remote training tool. Moreover, the application of DenTeach could be further extended to other fields of health sciences such as general surgery and neurosurgery where a drill is used to conduct a task.

## Data Availability Statement

The original contributions presented in the study and further inquiries can be directed to the corresponding author.

## Ethics Statement

The studies involving human participants were reviewed and approved by University of Alberta. The patients/participants provided their written informed consent to participate in this study.

## Author Contributions

LC drafted the work including literature review, explanation of the platform and test procedure, and contributed to data analysis. MK, AC, and AM performed the experiments, and contributed to data analysis. SM and MK provided technical support for dentistry teaching and learning, validated the results and findings, and checked the compatibility of results with real-world dentistry. All authors contributed to revising the manuscript and ensuring the correctness of the content. MT and YM provided funding for developing and validating the platform. MT contributed to the further editing of the manuscript and provided guidance and valuable suggestions/discussions.

## Funding

The Natural Sciences and Engineering Research Council (NSERC) of Canada under grant EGP 542825-19. The National Research Council Canada - Industrial Research Assistance Program (NRC-IRAP) under industrial grant 921303.

## Conflict of Interest

AM and YM work for the Department of Research and Development Tactile Robotics Company, the IP holder of the technology.

The remaining authors declare that the research was conducted in the absence of any commercial or financial relationships that could be construed as a potential conflict of interest.
